# Catheter ablation for treatment of patients with atrial fibrillation and heart failure: a meta-analysis of randomized controlled trials

**DOI:** 10.1186/s12872-018-0904-3

**Published:** 2018-08-13

**Authors:** Yingxu Ma, Fan Bai, Fen Qin, Yixi Li, Tao Tu, Chao Sun, Shenghua Zhou, Qiming Liu

**Affiliations:** Department of Cardiology, The Second Xiangya Hospital, Central South University, Changsha, Changsha, 410011 Hunan China

**Keywords:** Atrial fibrillation, Heart failure, Catheter ablation, Rhythm control strategy, Rate control strategy

## Abstract

**Background:**

There is a little evidence for the effects of catheter ablation (CA) on hard endpoints in patients with atrial fibrillation (AF) and heart failure (HF).

**Methods:**

PubMed, Embase and Cochrane Library were searched for randomized controlled trials (RCTs) enrolling patients with AF and HF who were assigned to CA, rate control or medical rhythm control groups. This meta-analysis was performed by using random-effect models.

**Results:**

Seven RCTs enrolling 856 participants were included in this meta-analysis. CA reduced the risks of all-cause mortality (risk ratio [RR] 0.52, 95% CI 0.35 to 0.76), HF readmission (RR 0.58, 95% CI 0.46 to 0.66) and the composite of all-cause mortality and HF readmission (RR 0.55, 95% CI 0.47 to 0.66) when compared with control. But there was no significant difference in cerebrovascular accident (RR 0.56, 95% CI 0.23 to 1.36) between two groups. Compared with control, CA was associated with improvement in left ventricular ejection fraction (mean difference [MD] 7.57, 95% CI 3.72 to 11.41), left ventricular end systolic volume (MD -14.51, 95% CI -26.84 to − 2.07), and left ventricular end diastolic volume (MD -3.78, 95% CI -18.51 to 10.96). Patients undergoing CA exhibited increased peak oxygen consumption (MD 3.16, 95% CI 1.09 to 5.23), longer 6-min walk test distance (MD 26.67, 95% CI 12.07 to 41.27), and reduced Minnesota Living with Heart Failure Questionnaire scores (MD -9.49, 95% CI -14.64 to − 4.34) than those in control group. Compared with control, CA was associated with improved New York Heart Association class (MD -0.74, 95% CI -0.83 to − 0.64) and lower B-type natriuretic peptide levels (MD -105.96, 95% CI -230.56 to 19.64).

**Conclusions:**

CA was associated with improved survival, morphologic changes, functional capacity and quality of life relative to control. CA should be considered in patients with AF and HF.

**Electronic supplementary material:**

The online version of this article (10.1186/s12872-018-0904-3) contains supplementary material, which is available to authorized users.

## Background

Atrial fibrillation (AF) often coexisted with heart failure (HF). HF could be observed in 10–50% of AF patients [1]. Meanwhile, up to 50% of HF patients had AF [2]. AF and HF shared some common risk factors, such as hypertension, diabetes, and ischemic cardiomyopathy. In addition, some identical pathophysiological mechanisms were involved in the development of AF and HF, including cardiac fibrosis and neurohumoral activation [3]. Loss of atrial contraction and rapid but irregular ventricular response impaired ventricular filling, which ultimately led to decreased cardiac output and left ventricular dysfunction [4]. Among HF patients, increased left ventricular filling pressure and atrial dilation gave rise to atrial electrical and structural remodelings, which consequently became the substrate for AF perpetuation [4]. The risks of all-cause death, cardiovascular death, HF hospitalization, and stroke in patients with AF and HF were far higher than those in patients with only HF. It’s noteworthy that differences between these two groups was independent of the types of AF [2].

For patients with AF and HF, antiarrhythmic treatments were classified to two strategies, rhythm control and rate control. AFFIRM trial [5] and AF-CHF trial [6] demonstrated that there was no difference in mortality between medical rhythm control and medical rate control groups. And AF-CHF trial [6] further indicated that AF hospitalization rate in rhythm control group was higher than that in rate control group. These two trials seemed to imply that rhythm control strategy was harmful in patients with AF and HF. However, failure to demonstrate superiority of rhythm control might be because of poor efficacy of antiarrhythmic drugs (AADs) in sinus rhythm maintenance and marked adverse effects of long-term AADs’ therapy. Recently, some observational studies [7] have shown sinus rhythm maintenance significantly improved cardiac function. Substudy of AFFIRM trial [5] also indicated that sinus rhythm was associated with improved survival. These studies implied that sinus rhythm maintenance might be beneficial to patients with AF and HF.

Catheter ablation (CA) is state-of-the-art rhythm control strategy for patients with non-permanent AF. CA without adverse effects of AADs was more effective than AADs in sinus rhythm maintenance [8]. Some observational studies, randomized controlled trials (RCTs), and meta-analyses [7, 9–13] demonstrated that CA improved cardiac function and exercise tolerance compared with rate control strategy. Therefore, the guideline [8] recommended CA should be considered in patients with AF and HF in order to relieve symptoms when tachycardiomyopathy was suspected (IIa, C). However, effects of CA on hard endpoints were unknown. Moreover, the meta-analysis [12] by Anselmino et al. quantitively synthesized the results of RCTs and small cohort studies, which was contradictory to recommendations of Cochrane Handbook for Systematic Reviews of Interventions (version 5.2). And the meta-analysis [13] by Halabi et al. enrolled only 224 patients and lacked comparisons of hard endpoints between two strategies, showing relatively limited clinical implications. Fortunately, CASTLE-AF trial [14] which was published this year investigated the effects of CA and medical therapy (rhythm control or rate control) on hard outcomes, providing evidence for strategy selection in patients with AF and HF. Hence, we sought to make a meta-analysis to evaluate efficacy and safety of CA in these patients, especially effects of CA on hard outcomes, aiming to offer cardiologists a more comprehensive picture of therapeutic strategies in patients with AF and HF.

## Methods

We performed this meta-analysis of RCTs according to recommendations from the guidelines of the Preferred Reporting Items for Systematic Reviews and Meta-Analyses (PRISMA) [15, 16] and Cochrane Handbook for Systematic Reviews of Interventions (version 5.2).

### Search strategy

Two investigators independently searched PubMed, Embase and Cochrane Library. The search strategy in PubMed incorporated the Cochrane Highly Sensitive Search Strategy [16]. We used following key words: “atrial fibrillation”, “heart failure”, and “catheter ablation” (details of search strategy included in Additional file 1).

### Selection criteria and quality assessment

RCTs enrolling patients with AF and HF who underwent CA were included. The studies included in this meta-analysis were published before February 27th 2018, in which language was restricted to English.

Cochrane collaboration’s tool for assessing risk of bias was used to assess the quality of included studies by two independent authors [16]. The items included in this tool were random sequence generation, allocation concealment, blinding of participants and personnel, blinding of outcome assessment, incomplete outcome data, and selective reporting.

### Data extraction and outcome measures

Two reviewers independently extracted data from included studies. Data extracted from studies included study characteristics, patient characteristics, details regarding CA and control groups, and outcome measures. Outcomes of interest for this meta-analysis were classified to procedural outcomes, hard outcomes, morphologic parameters, functional capacity and quality of life parameters, and symptom and biomarker parameters. Procedural outcomes included AF recurrence, AF burden, and procedural complication. AF burden was expressed as the percentage of total time in AF from time beyond blanking period. All-cause mortality, HF readmission, a composite of all-cause mortality and HF readmission, and cerebrovascular accident were of concern in hard outcomes. Morphologic parameters incorporated changes in left ventricular ejection fraction (LVEF), left atrial internal diameter, left ventricular end systolic volume (LVESV), and left ventricular end diastolic volume (LVEDV). We investigated peak oxygen consumption (VO_2_), 6-min walk test distance, and Minnesota Living with Heart Failure Questionnaire (MLWHF) scores in functional capacity and quality of life parameters. New York Heart Association (NYHA) class and B-type natriuretic peptide (BNP) levels were evaluated in symptom and biomarker parameters.

### Statistical analysis

This meta-analysis was performed by Review Manager 5.0 and Stata 12.0. Because CASTLE-AF trial [14] compared outcomes of CA and medical therapy (rhythm control or rate control) in patients with AF and HF,we quantitively synthesized RCTs in which rate control or medical rhythm control strategies were used in the control group to get extended interpretation of this meta-analysis results and implications for heterogeneous and complex clinical conditions. We performed two substudies. The first substudy quantitatively synthesized RCTs enrolling patients with persistent AF and HF. And the second one included RCTs comparing outcomes of CA and rate control strategies.

Outcome data were extracted as risk ratios (RRs) and 95% confidence intervals (CIs) or mean differences (MDs) and 95% CIs. Random-effect models were used for all outcomes due to the clinical heterogeneity of included studies. The Cochran’s Q test and I^2^ test were performed to assess the heterogeneity of the summary effects. If the *P* value of Cochran’s Q test was < 0.10 and I^2^ was > 50%, heterogeneity was considered to exist. Publication bias were assessed by funnel plot, Begg’s test and Egger’s test, respectively.

To further detect clinical heterogeneity, sensitivity analysis was performed for LVEF change and a composite of all-cause mortality and HF readmission. We included trials in which AF freedom rate was > 80% at the end of follow-up.

## Results

The results of study selection process were shown in Additional file 1: Figure S1. Of 2803 articles found after the search process, 7 RCTs were included in this meta-analysis [9–11, 14, 17–19].

### Characteristics of studies and patients

Seven studies involving 856 patients were included in our meta-analysis. Mean age ranged from 55 to 64 years old. LVEF of all the patients was < 50% and NYHA class was II-IV. Two trials [9, 14] enrolled paroxysmal and persistent AF patients, whereas only persistent AF patients were included in another five trials [10, 11, 17–19]. All the participants in CA group underwent pulmonary vein isolation (PVI), most of who underwent additional linear and complex or fractionated electrograms ablation. Medical rate control strategy was introduced to control group in 4 trials [10, 11, 17, 19] while atrioventricular-node ablation with biventricular pacing was performed in PABA-CHF trial [9]. AATAC trial [18] used amiodarone to achieve rhythm control and patients in the control group of CASTLE-AF trial [14] accepted medical treatment (rhythm control or rate control). Further characteristics of included studies and patients were summarized in Additional file 1: Table S1 and S2, respectively.

According to Cochrane collaboration’s tool for assessing risk of bias,the quality of included studies was relatively high (Additional file 1: Figure S2). Effect sizes reported in studies were distributed symmetrically (Additional file 1: Figure S3) and there was no significant bias from small studies (Begg’s test *P* = 1.00; Egger’s test *P* = 0.31), indicating that publication bias was low.

### Procedural outcomes

Among 429 patients undergoing CA in the included trials, 64.2% (95% CI 49.4% to 79.0%) were free from AF after initial procedure. CA was repeated in patients with recurrent AF during follow-up. At the end of follow-up, 74.9% (95% CI 63.2% to 86.5%) of patients in the CA group had freedom from AF and AF burden was 14.2% (95% CI -10.7% to 39.1%). Procedural complication rate was 6.4% (95% CI 2.7% to 10.1%).

### Mortality, readmission, cerebrovascular accident

**Fig. 1:**
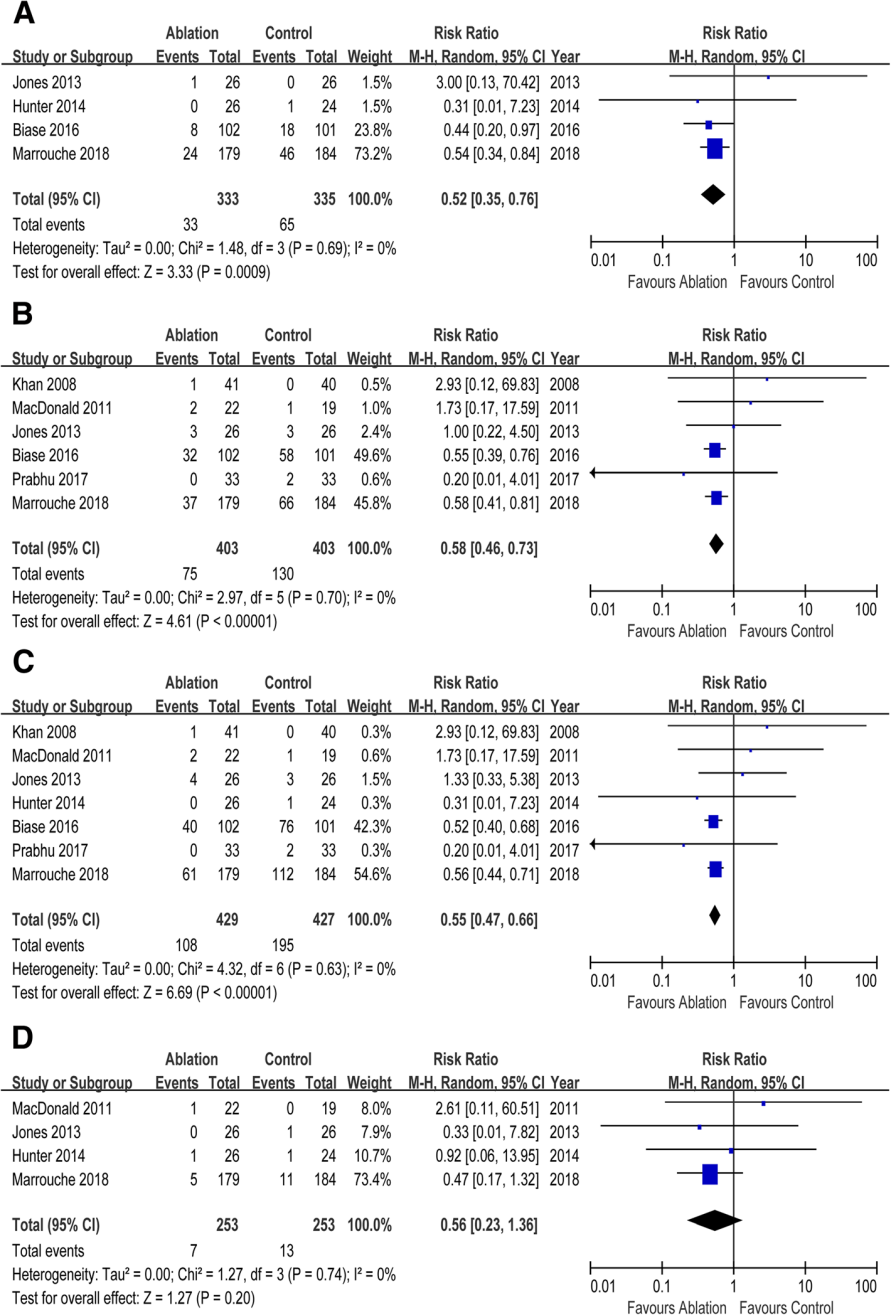
Forest plot with individual and summary estimates of the risk ratio (RR) and 95% confidence interval (CI) of mortality, readmission, cerebrovascular accident in this main analysis. (**a**) All-cause mortality. (**b**) HF readmission. (**c**) Composite of all-cause mortality and HF readmission. **(d)** Cerebrovascular accident. CI, confidence interval

Compared with control, CA markedly reduced risks of all-cause mortality (RR 0.52, 95% CI 0.35 to 0.76) and HF readmission (RR 0.58, 95% CI 0.46 to 0.66). And CA was associated with decreased risk of the composite of all-cause mortality and HF readmission (RR 0.55, 95% CI 0.47 to 0.66). But no difference was observed in cerebrovascular accident between two groups (RR 0.56, 95% CI 0.23 to 1.36). (Fig. 1).

### Morphologic changes

**Fig. 2:**
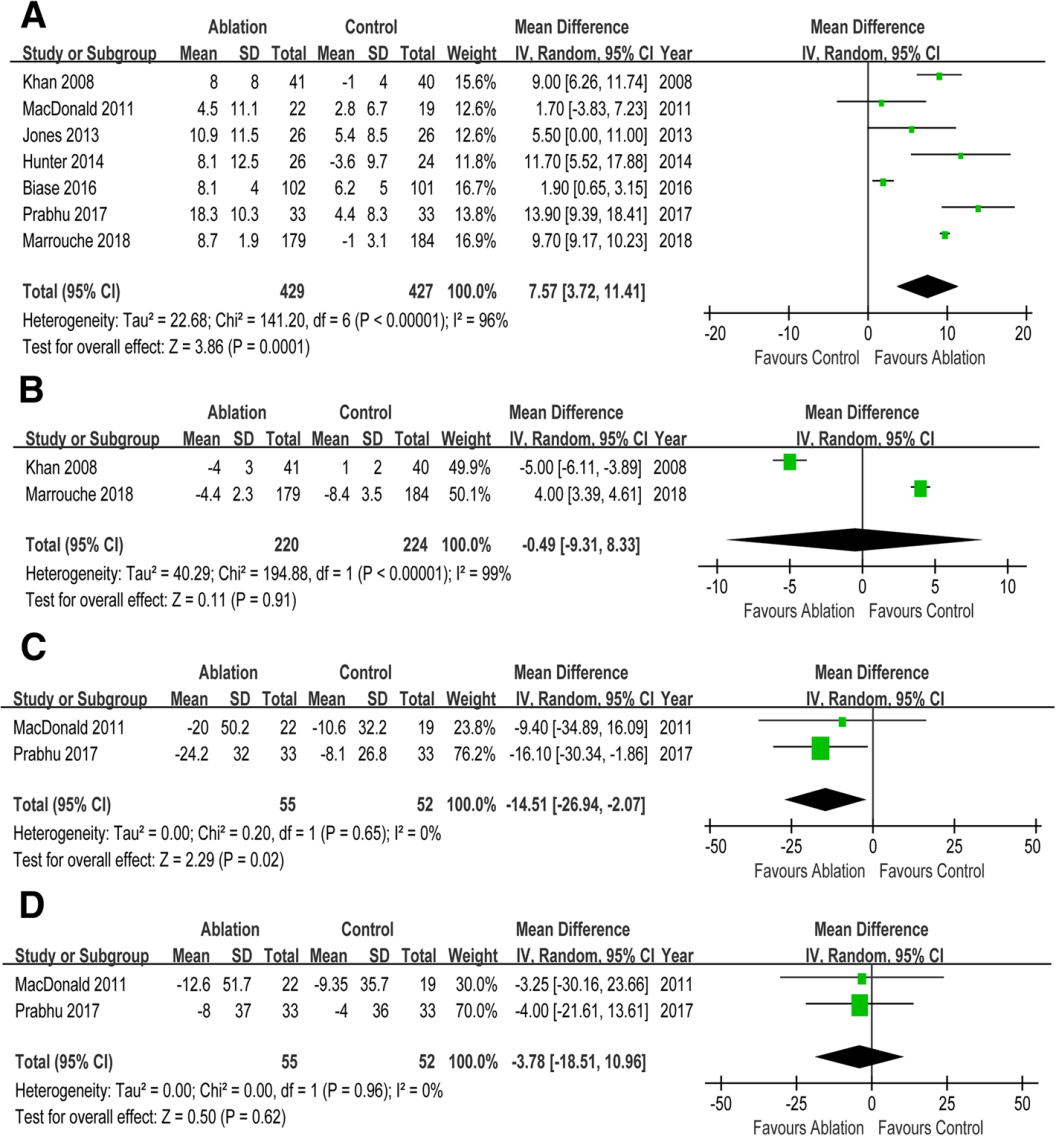
Forest plot with individual and summary estimates of the mean difference (MD) and 95% confidence interval (CI) of morphologic changes. (**a**) Change in LVEF. (**b**) Change in left atrial internal diameter. (**c**) Change in LVESV. (**d**) Change in LVEDV. SD, standard deviation

When compared with control, CA was associated with a 7.57% increase in LVEF (MD 7.57, 95% CI 3.72 to 11.41). There was no difference in left atrial internal diameter between two groups (MD -0.49, 95% CI -9.31 to 8.33). CA was associated with decreased LVESV (MD -14.51, 95% CI -26.94 to − 2.07). And CA reduced LVEDV with a slight trend toward significance (MD -3.78, 95% CI -18.51 to 10.96). (Fig. 2).

### Functional capacity and quality of life

**Fig. 3:**
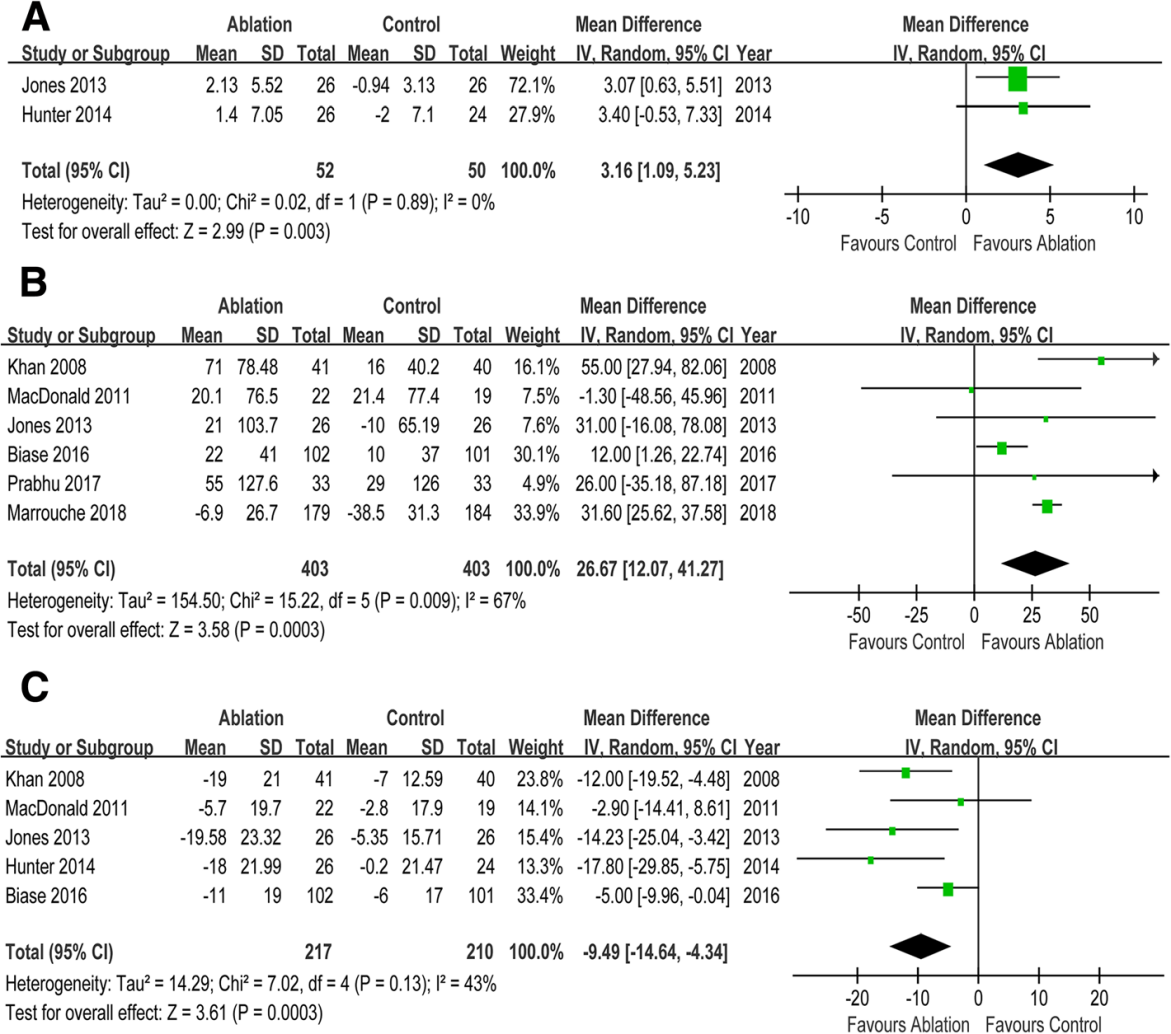
Forest plot with individual and summary estimates of the mean difference (MD) and 95% confidence interval (CI) of functional capacity and quality of life. (**a**) Change in peak VO_2_. (**b**) Change in 6-min walk test distance. (**c**) Change in MLWHF scores. SD, standard deviation

Compared with control, CA was associated with a 3.16 ml/kg/min increase in peak VO_2_ (MD 3.16, 95% CI 1.09 to 5.23). There was a significant improvement in 6-min walk test distance in CA group versus that in control group (MD 26.67, 95% CI 12.07 to 41.27). Marked reduction in MLWHF scores was observed in patients undergoing CA compared with control (MD -9.49, 95% CI -14.64 to − 4.34). (Fig. 3).

### Symptom and biomarker

**Fig. 4:**
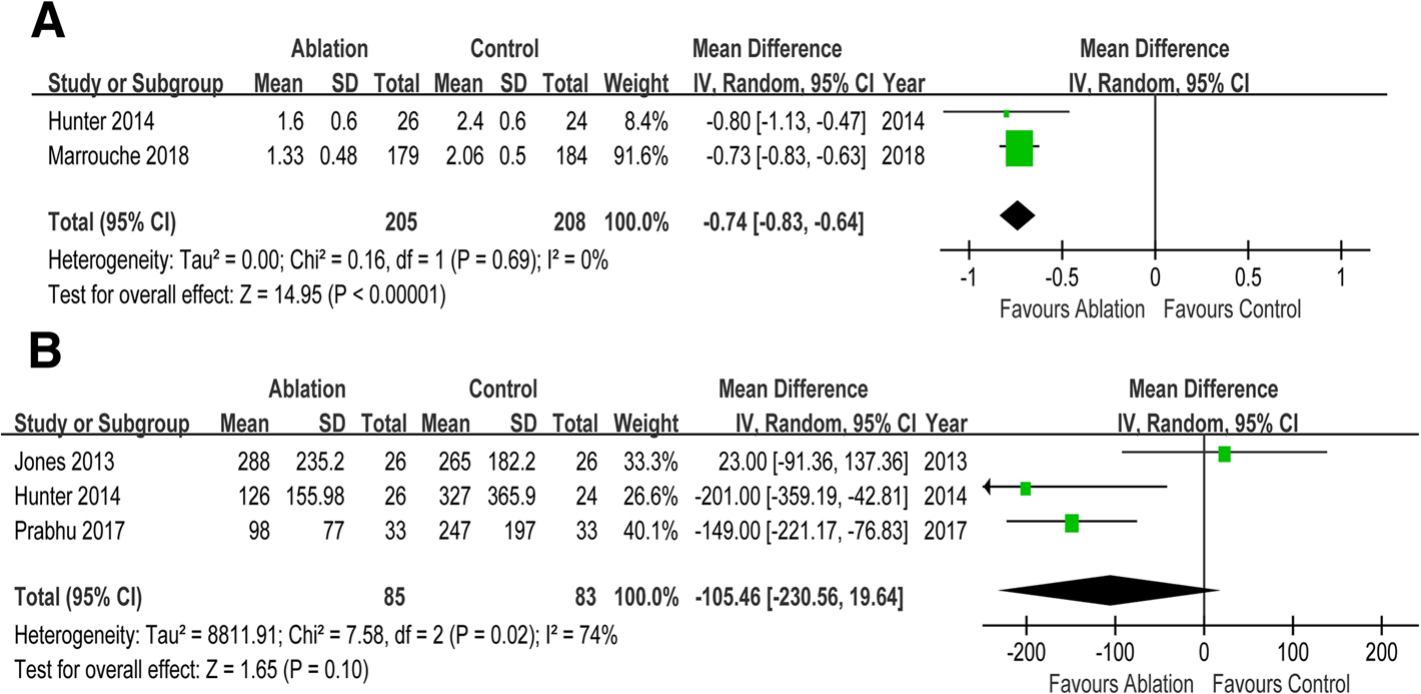
Forest plot with individual and summary estimates of the mean difference (MD) and 95% confidence interval (CI) of symptom and biomarker. (**a**) NYHA scores. (**b**) BNP. SD, standard deviation

Compared with control, patients in CA group had lower NYHA class (MD -0.74, 95% CI -0.83 to − 0.64). And BNP levels were lower in CA group than that in control group (MD -105.46, 95% CI -230.56 to 19.64). (Fig. 4).

### Substudy for patients with persistent AF and HF

5 trials [10, 11, 17–19] enrolled patients with persistent AF and HF, comparing outcomes of CA and medical therapy (rhythm control or rate control). Among 412 patients included in this subgroup analysis, 209 patients underwent CA. After the initial procedure, 60.1% (95% CI 42.2% to 78.0%) of patients were in sinus rhythm. For patients with recurrent AF, CA was repeated. And sinus rhythm was observed in 74.6% (95% CI 59.1% to 90.2%) of patients at the end of follow-up. CA reduced risks of all-cause mortality (RR 0.48, 95% CI 0.23 to 1.01), HF readmission (RR 0.57, 95% CI 0.41 to 0.78), and the composite of all-cause mortality and HF readmission (RR 0.54, 95% CI 0.42 to 0.70) relative to medical therapy. (Additional file 1: Figure S4) CA was associated with a 6.76% increase in LVEF (MD 6.76, 95% CI 1.42 to 12.11). (Additional file 1: Figure S5) When compared with medical therapy, significant improvement in 6-min walk test distance (MD 12.56, 95% CI 2.56 to 22.73) and MLWHF scores (MD -8.96, 95% CI -15.50 to − 2.43) was observed in CA group. (Additional file 1: Figure S6).

### Substudy comparing CA and rate control strategy

Endpoints of CA and rate control groups were compared in 5 RCTs [9–11, 17, 19]. A total of 290 participants was enrolled in this substudy, among who 148 patients underwent CA. There were no differences in all-cause mortality (RR 0.96, 95% CI 0.10 to 8.95), HF readmission (RR 1.04, 95% CI 0.35 to 3.09), and the composite of all-cause mortality and HF readmission (RR 1.06, 95% CI 0.39 to 2.87) between two groups. (Additional file 1: Figure S7) When compared with rate control, CA was associated with an improvement in LVEF (MD 8.50, 95% CI 4.66 to 12.34), 6-min walk test distance (MD 33.33, 95% CI 7.38 to 59.29), and MLWHF scores (MD -11.75, 95% CI -17.14 to − 6.36). (Additional file 1: Figure S8 and S9).

### Sensitivity analysis

In 4 trials [9, 11, 17, 19], AF freedom rate of patients undergoing CA was > 80% at the end of follow-up. Rate control strategy was introduced to control group in these trials. No difference was observed in the composite of all-cause death and HF readmission between two groups (RR 0.95, 95% CI 0.32 to 2.86). CA increased LVEF (MD 10.00, 95% CI 6.76 to 13.25) compared to rate control.

## Discussion

We made the first meta-analysis of RCTs which concentrated on effects of CA on hard outcomes in patients with AF and HF. We found that AF ablation was effective and safe in these patients. CA reduced risks of all-cause mortality and HF readmission, but it did not prevent cerebrovascular accident. CA was related to increased LVEF and reduced left ventricular volume, which consequently resulted in better cardiac function. Meanwhile, CA relieved HF symptoms, increased exercise tolerance, and improved quality of life.

Most of patients (81.2%) included in this meta-analysis presented with persistent AF. The proportion of patients in sinus rhythm after the initial procedure was relatively low but redo procedure increased the proportion of patients free from AF, which was consistent with results in STAR AF II trial [20] enrolling patients without HF. This might imply that the presence of HF did not lower efficacy of AF ablation. Moreover, the results indicated that repeated procedure should be considered in HF patients with recurrent AF to achieve sinus rhythm. Although STAR AF II trial [20] showed that there was no difference between PVI and PVI plus additional ablation in patients without HF, PVI plus additional ablation (linear or complex fractionated electrograms) might be beneficial in patients with AF and HF. Because co-occurrence of AF and HF brought about large atrial stress and severe structural and electrical remodeling [1]. This needed to be confirmed by large RCTs. The presence of HF made heart structure viable [1], increasing the procedural difficulty. But this meta-analysis showed that procedural complication risk was relatively low, indicating that ablation was safe in HF patients.

Previous meta-analyses [12, 13] demonstrated only soft endpoints of CA in patients with AF and HF. These studies indicated that CA improved LVEF and exercise tolerance. However, the number of patients enrolled in these studies was small and they did not report the effects of CA on hard outcomes, such as mortality and HF readmission. We presented the first meta-analysis to evaluate the impact of CA on hard endpoints. We quantitively synthesized trials including rate control strategy (medicine or device assistance) and AADs in the main analysis because previous studies [6, 21] have not shown one strategy or drug to be superior to another.

Previous studies [6, 21] suggested that sinus rhythm maintenance by AADs did not lower mortality risk in patients with AF and HF. But this might be because the adverse effects of AADs (amiodarone [6] and dofetilide [21]) were common and serious and the harm exceeded the benefits after long-term use. Our meta-analysis indicated sinus rhythm achieved by CA reduced risks of all-cause mortality and HF readmission. Results of CABANA trial presented in 2018 HRS were consistent with our analysis. This trial showed CA reduced mortality or cardiovascular hospitalization (HR 0.83, 95% CI 0.74 to 0.93) by 17% compared to medical therapy. According to the actual treatment that patients accepted, CA was superior to drug therapy in the primary endpoint (HR 0.67, 95% CI 0.50 to 0.89). The rates of all-cause mortality (4.4% vs. 7.5%) and cardiovascular hospitalization (41.2% vs. 74.9%) in CA group were lower than those in medical therapy group. These results all supported that sinus rhythm achieved through CA was beneficial to patients in terms of hard outcomes.

Notably, the risks of all-cause mortality, HF readmission, and the composite of all-cause mortality and HF readmission in the substudy enrolling patients with persistent AF and HF were slightly lower than those in the main analysis. This might be because the main analysis included paroxysmal AF patients (18.8%) besides persistent AF patients. And Mogensen et al. [2] found that paroxysmal AF patients were at greater risks of mortality and HF readmission than persistent AF patients. The underlying mechanism was uncertain, but it’s possible that paroxysms of AF reflected HF instability. Our result implied that patients with persistent AF and HF were easier to benefit from CA.

Our meta-analysis failed to demonstrate protection of CA on cerebrovascular accident. This was because patients with AF and HF had a higher CHA_2_DS_2_-VASc score. The presence of HF in AF patients increased stroke risk by 0.25–1.27 [2]. Thus, guideline recommended that patients with high CHA_2_DS_2_-VASc scores should accept anticoagulation therapy even though they were in sinus rhythm [8].

Substudy comparing outcomes of CA and rate control strategies did not find the advantages of CA on all-cause mortality and HF readmission. But this did not mean that sinus rhythm achieved by CA was not superior to rate control strategy. Because there were some drawbacks in this substudy, which extremely limited interpretation of the substudy results. Firstly, the number of patients enrolled in this substudy was a bit small. For example, only 102 patients were included in the comparison of all-cause mortality. Secondly, the duration of follow-up of included studies was short (6–12 months). Nevertheless, CASTLE-AF trial [14] indicated mortality benefit of ablation did not emerge until after 36 months. Thus, we expected the long-term results of these included studies [9–11, 17, 19] to make a thorough evaluation. Furthermore, this substudy demonstrated that CA was better than rate control strategy in terms of soft endpoints. For improved cardiac function and quality of life, CA should be performed rather than rate control strategy.

This meta-analysis seemed to show that CA did not significantly reduce left atrial internal diameter when compared with control. Yet, two trials [9, 14] included in this comparison showed that left atrial internal diameter in patients undergoing CA was reduced (*P* < 0.05) from baseline to the end of follow-up. This suggested CA was related to reduced atrial internal diameter, albeit not superior over rate control or medical rhythm control. Regression of left atrial volume contributed to less AF recurrence [11]. In addition, our analysis demonstrated CA decreased left ventricular volume. Reduced left ventricular volume led to less ventricular stress, improved ventricular filling, protection against fibrosis, and prevention of lung function damage [22], which had a positive impact on prognosis. We found CA increased LVEF, but it’s controversial about the role of LVEF in the prognosis prediction. Because the measure of LVEF was influenced by many factors and repeatability of existing measure methods was poor [11], especially under AF rhythm. Our study also demonstrated maintenance of sinus rhythm increased peak VO_2_, improved MLWHF scores, and lowered BNP levels. Previous studies [11, 13] suggested that these three parameters were objective and improvement in these parameters was associated with better survival.

### Limitations

This meta-analysis lacked specified individual data to conduct subgroup analyses based on age, sex, and baseline diseases. But the heterogeneity of most analyses was small and there was no need to explore the source of heterogeneity. In addition, baseline LVEF was measured under AF rhythm in the included studies and echocardiography was used in 4 trials [9, 11, 17, 18], which meant relatively low accuracy and poor repeatability. However, LVEF measure at the end of follow-up was relatively accurate because most participants in CA group were in sinus rhythm. This might bring about errors in the comparison from baseline to the end of follow-up.

## Conclusions

CA was associated with reduced risks of all-cause mortality and HF readmission and improvement in functional capacity, exercise tolerance, and quality of life relative to control. CA might be related to better prognosis and prolonged survival, which still needed to be confirmed by the large RCT.

## Additional file

Additional file 1: Supplement Materials. (DOCX 2246 kb)

## Abbreviation

AAD: Antiarrhythmic drug
AF: Atrial fibrillation
BNP: B-type natriuretic peptide
CA: Catheter ablation
CI: Confidence interval
HF: Heart failure
LVEDV: Left ventricular end diastolic volume
LVEF: Left ventricular ejection fraction
LVESV: Left ventricular end systolic volume
MD: Mean difference
MLWHF: Minnesota Living with Heart Failure Questionnaire
NYHA: New York Heart Association
RCT: Randomized controlled trial
RR: Risk ratio
VO_2_: Peak oxygen consumption
