# Supplementary material for: Catheter ablation for treatment of patients with atrial fibrillation and heart failure: a meta-analysis of randomized controlled trials
Source: BMC Cardiovasc Disord. 2018 Aug 13;18:165. doi: 10.1186/s12872-018-0904-3 (PMC6090632; doi:10.1186/s12872-018-0904-3)
Supplement: Supplementary file 1 — Supplement Materials. (DOCX 2246 kb) [file 12872_2018_904_MOESM1_ESM.docx]

**Supplementary Data**

**Supplementary Methods**

**Search Strategy**

PubMed database

#1 “atrial fibrillation”

#2 “heart failure” OR “cardiac failure” OR “myocardial failure” OR “reduced left ventricular function” OR “systolic dysfunction” OR “diastolic dysfunction”

#3 ablation OR "Catheter Ablation"[Mesh] OR “radiofrequency ablation” OR “radiofrequency catheter ablation” OR “catheter radiofrequency ablation”

#4 #1 AND #2 AND #3

Embase

#1 'atrial fibrillation'

#2 'heart failure' OR 'congestive heart failure therapy' OR 'cardiac failure' OR 'myocardial failure' OR 'reduced left ventricular function' OR 'systolic dysfunction' OR 'diastolic dysfunction' OR 'systolic heart failure' OR 'diastolic heart failure'

#3 ablation OR 'ablation therapy' OR 'catheter ablation' OR 'radiofrequency ablation' OR 'radiofrequency catheter ablation' OR 'catheter radiofrequency ablation'

#4 #1 AND #2 AND #3

Cochrane Library

#1 “atrial fibrillation”

#2 “heart failure” OR “cardiac failure” OR “myocardial failure” OR “reduced left ventricular function” OR “systolic dysfunction” OR “diastolic dysfunction”

#3 ablation OR "catheter ablation" OR “radiofrequency ablation” OR “radiofrequency catheter ablation” OR “catheter radiofrequency ablation”

#4 #1 AND #2 AND #3


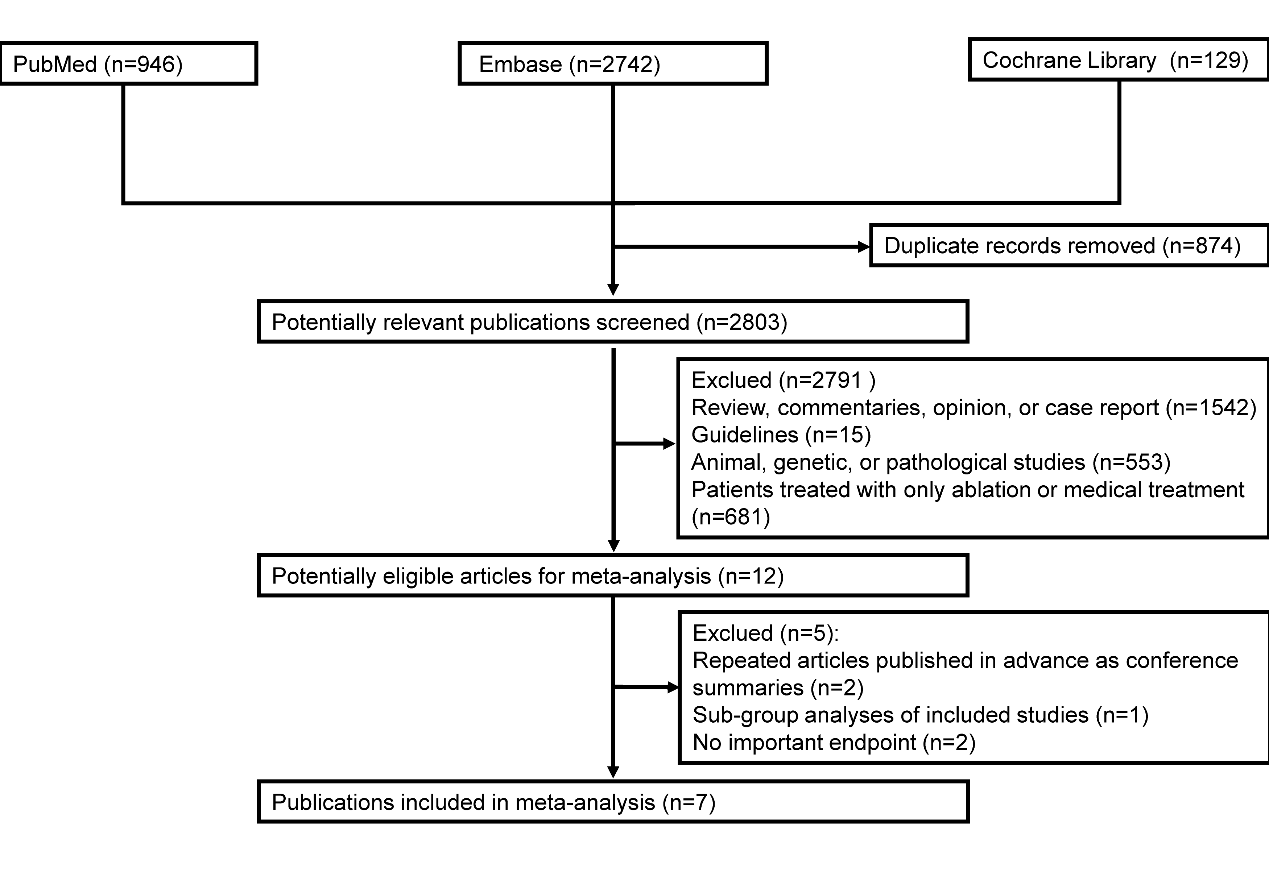


**Supplementary Figure S1** Flow chart showing the process of study selection and numbers of studies included.


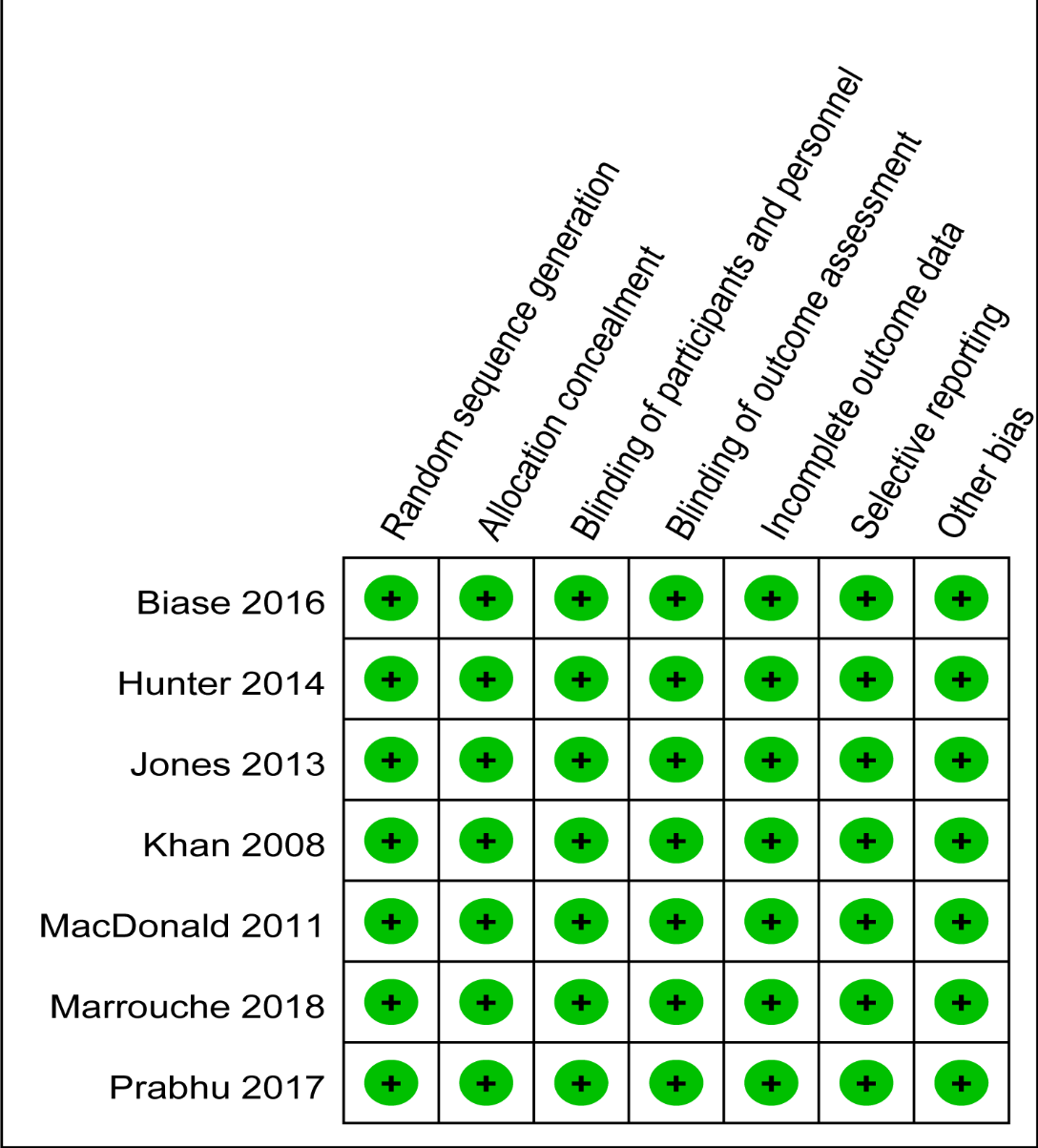


**Supplementary Figure S2** Risk of reporting bias for each study included in this meta-analysis.


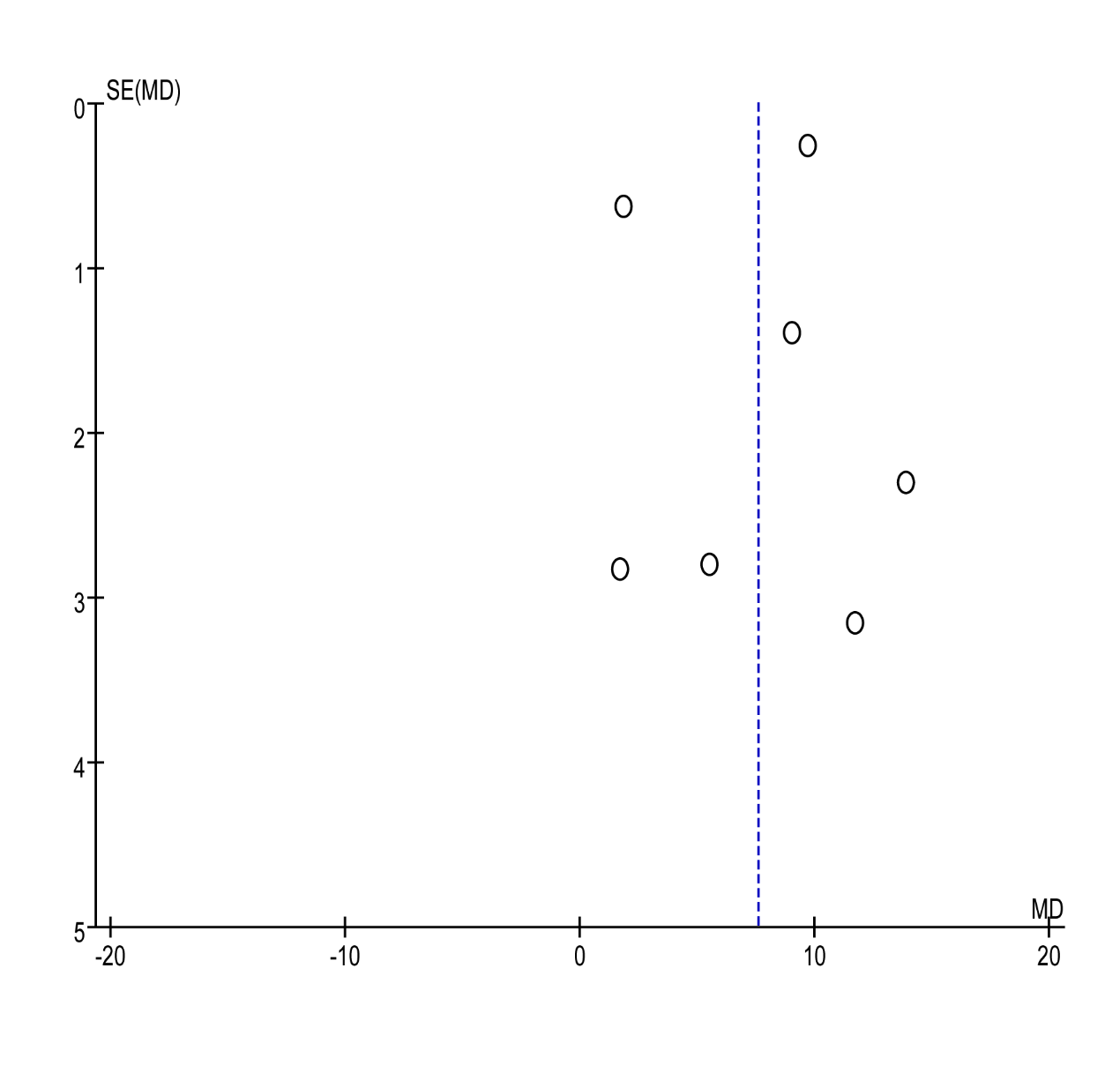


**Supplementary Figure S3** Assessment of publication bias by the funnel plot.

MD, mean difference; SE, standard error.


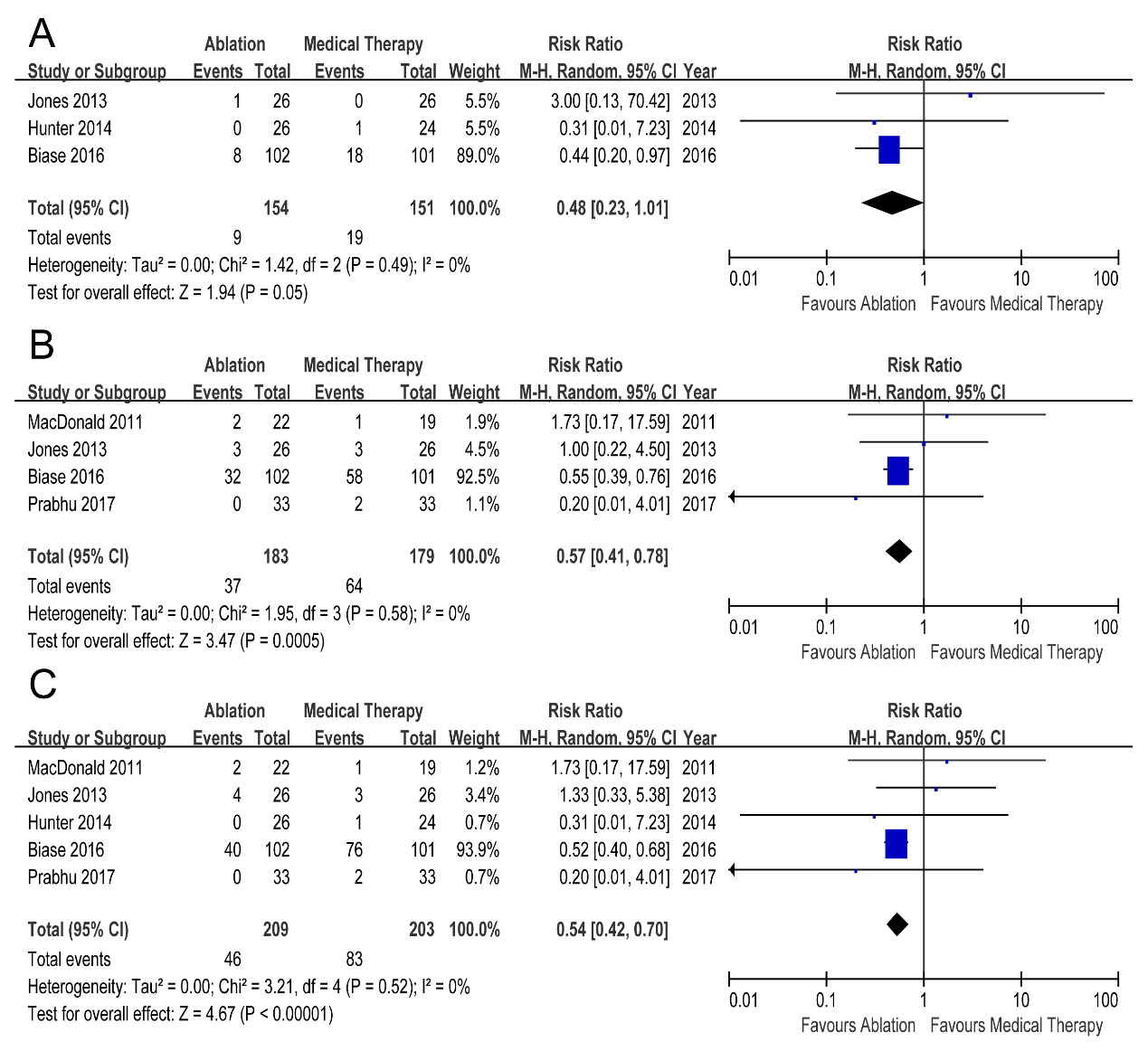


**Supplementary Figure S4** Forest plot with individual and summary estimates of the risk ratio (RR) and 95% confidence interval (CI) of mortality, readmission, composite of mortality and readmission.

**(A)** All-cause mortality. **(B)** HF readmission. **(C)** Composite of all-cause mortality and HF readmission.

CI, confidence interval.


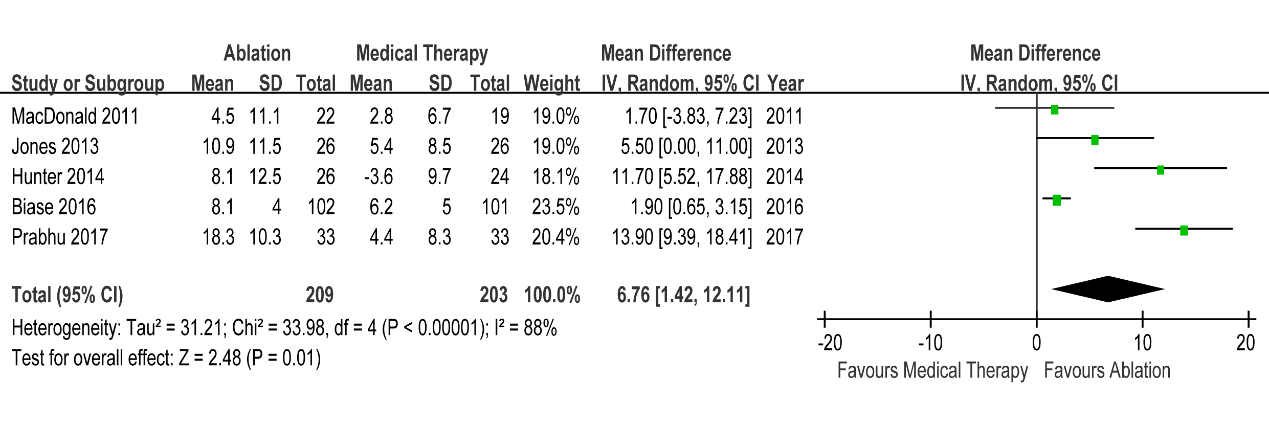


**Supplementary Figure S5** Forest plot with individual and summary estimates of the mean difference (MD) and 95% confidence interval (CI) of change in LVEF.

SD, standard deviation.


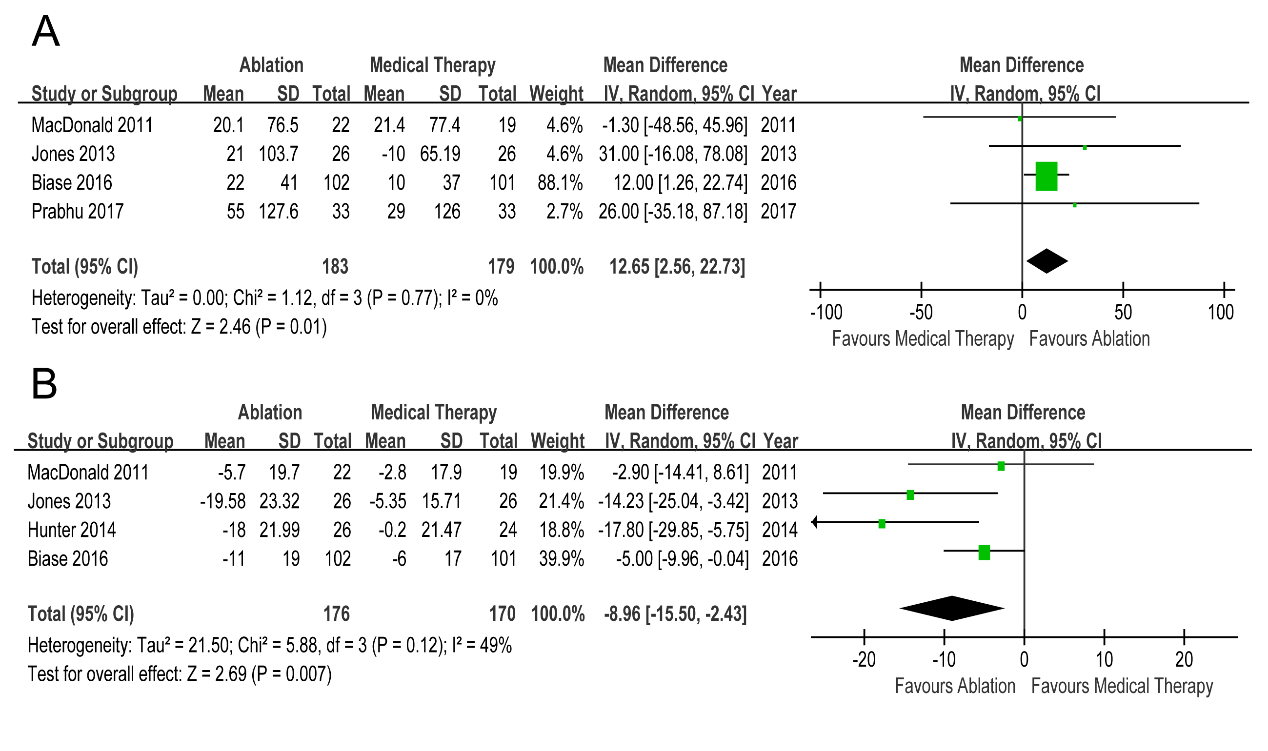


**Supplementary Figure S6** Forest plot with individual and summary estimates of the mean difference (MD) and 95% confidence interval (CI) of functional capacity and quality of life.

**(A)** Change in 6-min walk test distance. **(B)** Change in MLWHF scores.

SD, standard deviation.


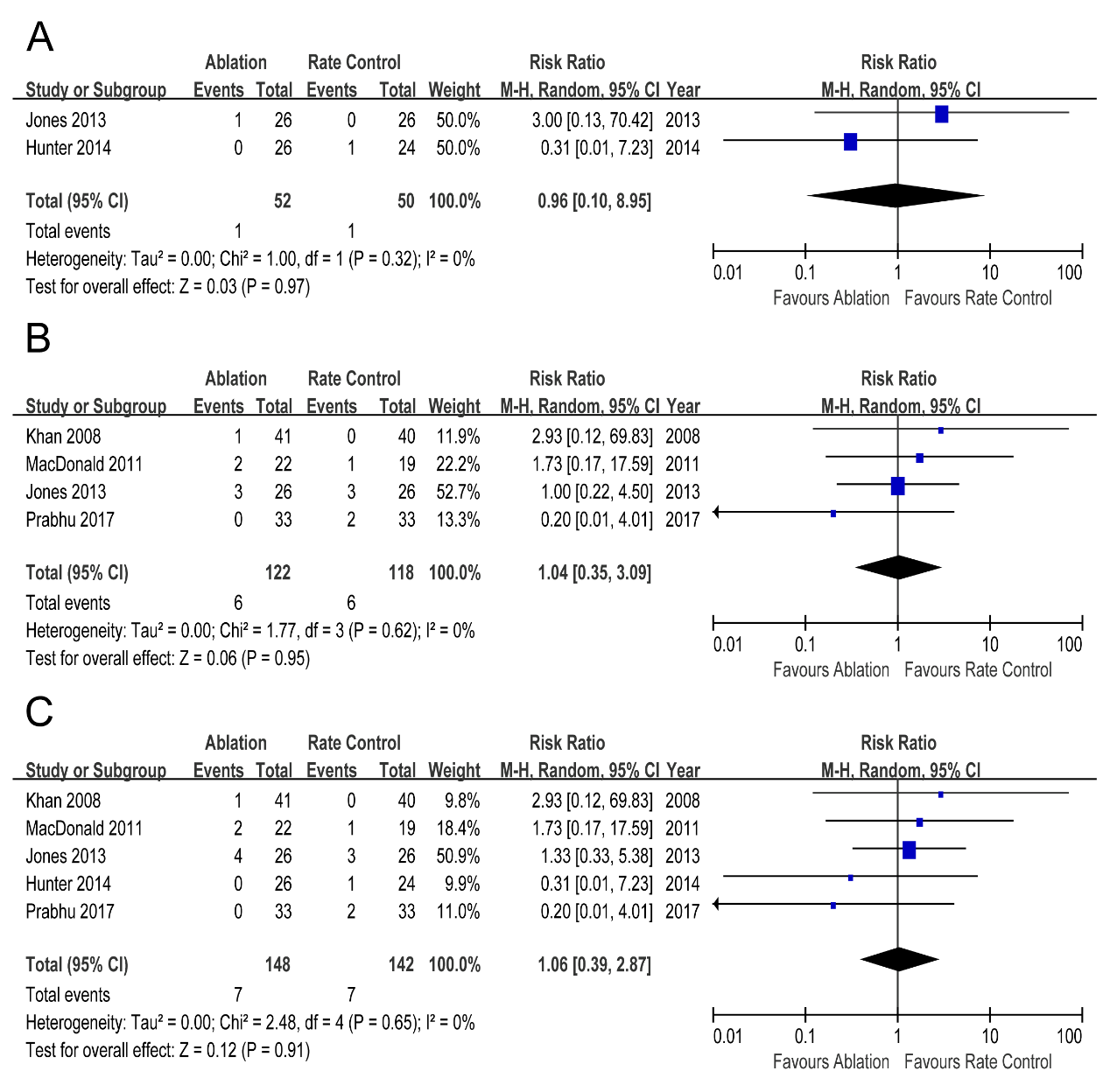


**Supplementary Figure S7** Forest plot with individual and summary estimates of the risk ratio (RR) and 95% confidence interval (CI) of mortality, readmission, composite of mortality and readmission.

**(A)** All-cause mortality. **(B)** HF readmission. **(C)** Composite of all-cause mortality and HF readmission.

CI, confidence interval.


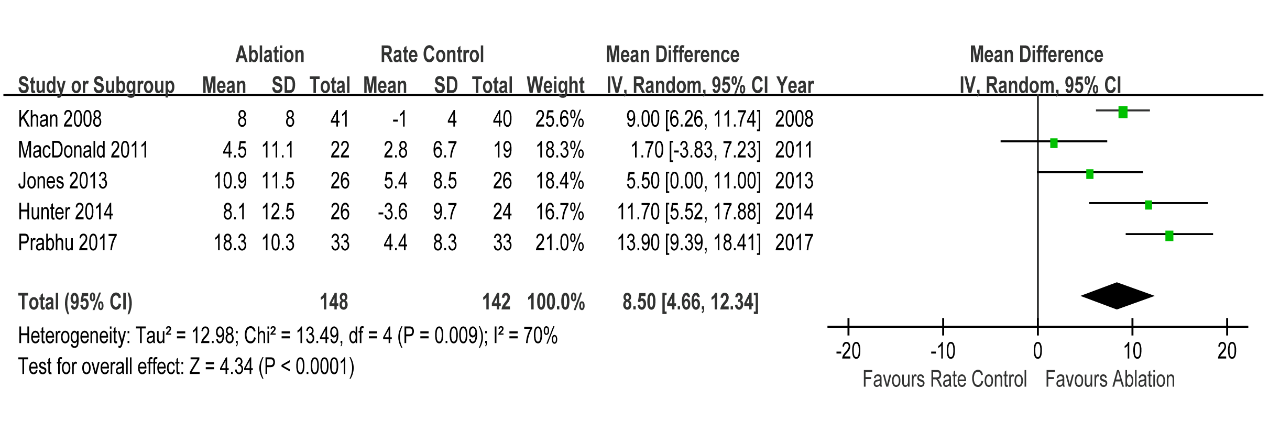


**Supplementary Figure S8** Forest plot with individual and summary estimates of the mean difference (MD) and 95% confidence interval (CI) of change in LVEF.

SD, standard deviation.


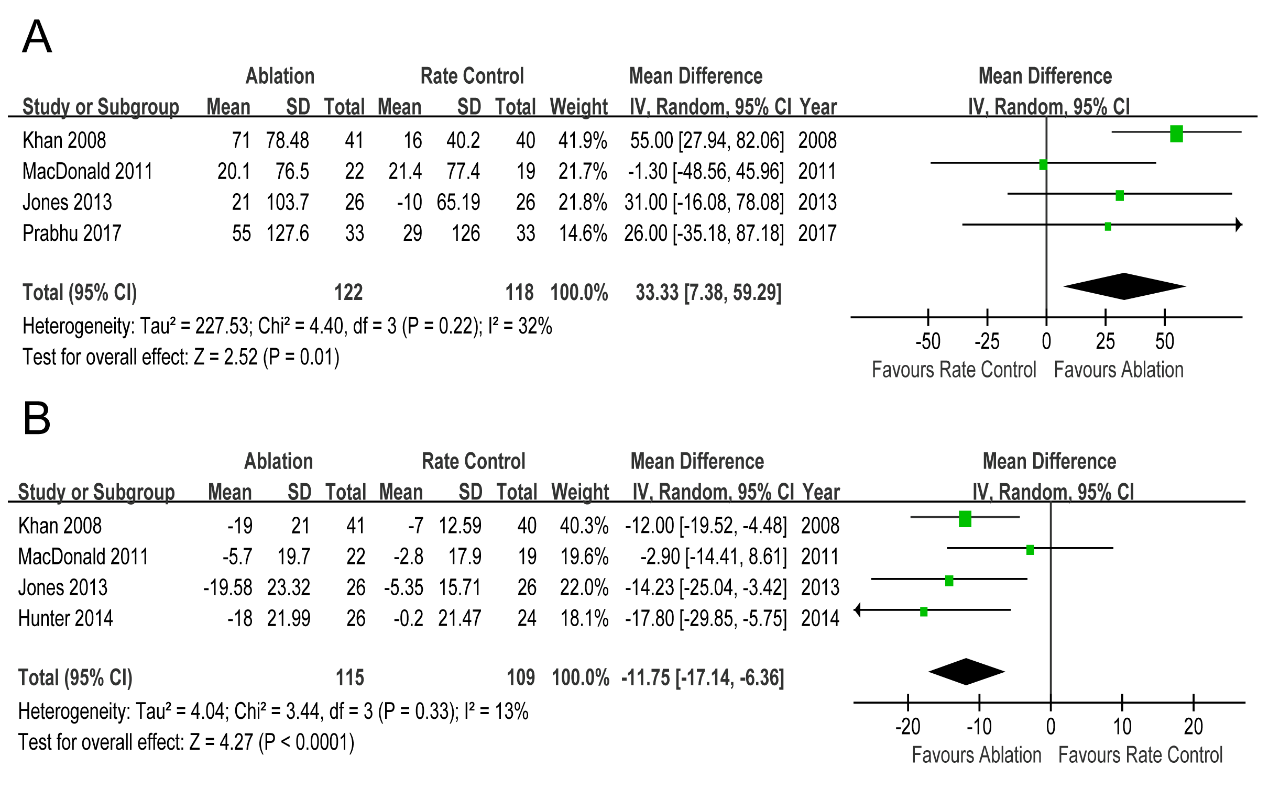


**Supplementary Figure S9** Forest plot with individual and summary estimates of the mean difference (MD) and 95% confidence interval (CI) of functional capacity and quality of life.

**(A)** Change in 6-min walk test distance. **(B)** Change in MLWHF scores.

SD, standard deviation.

**Supplementary Table S1** Characteristics of the included randomized controlled trials.

| First author, year of publication | N. patients | Follow-up (months) | Inclusion criteria | Ablation strategy | Control group strategy | SR following first procedure (%) | SR at follow-up end (%) |
| --- | --- | --- | --- | --- | --- | --- | --- |
| Khan, 2008 (1) | 81 | 6 | Symptomatic AF, NYHA Ⅱ or Ⅲ HF, LVEF≤40% | Pulmonary vein isolation, ablation of additional linear lesions and the sources of complex fractionated electrograms according to the preference of the center | Atrioventricular-node ablation with biventricular pacing | 78 | 88 |
| MacDonald, 2011 (2) | 41 | 6 | Persistent AF, NYHA Ⅱ-Ⅳ, LVEF<35% | Pulmonary vein isolation, linear lesion and complex fractionated electrogram ablation if needed | optimal HF treatment, digoxin added if mean HR>80 bpm over 24 hours | 50 | 50 |
| Jones, 2013 (3) | 52 | 12 | Persistent AF, symptomatic HF, NYHA Ⅱ-Ⅳ, LVEF≤35% | Pulmonary vein isolation, linear ablation, and ablation of complex fractionated electrograms | beta-blockers and/or digoxin achieve a mean HR≤80 bpm at rest and ≤110 bpm after a 6-min walk | 72 | 92 |
| Hunter, 2014 (4) | 50 | 6 | Persistent AF, symptomatic HF, NYHA Ⅱ-Ⅳ, LVEF<50% | Pulmonary vein isolation, ablation of complex fractionated electrograms, and linear ablation | β-blockers, ACEI, or ARB, and in selected patients spironolactone (if NYHA class ≥III and LVEF <35%) | 38 | 81 |
| Biase, 2016 (5) | 203 | 24 | Persistent AF, dual-chamber ICD or CRT-D implantation, NYHA Ⅱ or Ⅲ, LVEF≤40% | Pulmonary vein isolation, ablation of complex fractionated electrograms, linear ablation, and superior vena cava ablation if needed | Amiodarone | N/A | 70 |
| Prabhu, 2017 (6) | 66 | 6 | Persistent AF, NYHA Ⅱ-Ⅳ, LVEF≤40%, significant coronary artery disease excluded, no other identifiable cause explaining the left ventricular dysfunction | Pulmonary vein isolation, and linear ablation | Medical therapy titrated to achieve a resting rate <80 bpm, an average 24-h ventricular rate <100 bpm, and a post-exercise rate <110 bpm in accordance with current guidelines | 75 | 100 |
| Marrouche, 2018 (7) | 363 | 60 | Paroxysmal or persistent AF, NYHA Ⅱ-Ⅳ HF, | Pulmonary vein isolation, and additional ablation at the discretion of the operators | Medical rhythm or rate control strategy. The aim of rate control was a ventricular rate of 60 to 80 bpm at rest and 90 to 115bpm during moderate exercise | N/A | 63.1 |

ACEI, angiotensin converting enzyme inhibitor; AF, atrial fibrillation; ARB, angiotensin receptor inhibitor; bpm, beats per minute; CRT-D, cardiac resynchronization therapy defibrillator; HF, heart failure; HR, heart rate; ICD, implantable cardioverter defibrillator; LVEF, left ventricular ejection fraction; NYHA, New York Heart Association; SR, sinus rhythm.

**Supplementary Table S2** Patient Demographics.

|  | Khan et al. (1), 2008 | | MacDonald et al. (2), 2011 | | Jones et al. (3), 2013 | | Hunter et al. (4), 2014 | |
| --- | --- | --- | --- | --- | --- | --- | --- | --- |
|  | Ablation (n=41) | Rate control* (n=40) | Ablation (n=22) | Medical rate control (n=19) | Ablation (n=26) | Medical rate control (n=26) | Ablation (n=26) | Medical rate control (n=24) |
| Age, yrs | 60 ± 8 | 61 ± 8 | 62.3 ± 6.7 | 64.4 ± 8.3 | 64 ± 10 | 62 ± 9 | 55 ± 12 | 60 ± 10 |
| Male, n (%) | 39 (95) | 35 (88) | 17 (77) | 15 (79) | 21 (81) | 24 (92) | 25 (96) | 23 (96) |
| Body mass index, kg/m^2^ | N/A | N/A | 30 ± 5.6 | 30 ± 5.7 | N/A | N/A | N/A | N/A |
|  |  |  |  |  |  |  |  |  |
| Type of AF, n (%) |  |  |  |  |  |  |  |  |
| Paroxysmal | 20 (49) | 22 (55) | 0 (0) | 0 (0) | 0 (0) | 0 (0) | 0 (0) | 0 (0) |
| Persistent or long standing persisitent | 20 (51) | 18 (45) | 22 (100) | 19 (100) | 26 (100) | 26 (100) | 26 (100) | 24 (100) |
|  |  |  |  |  |  |  |  |  |
| Duration of AF, months | 48 ± 28.8 | 46.8 ± 33.6 | 44 ± 36.5 | 64 ± 47.6 | 23 ± 22 | 24 ± 29 | 24 (17-33) | 24 (12-48) |
|  |  |  |  |  |  |  |  |  |
| Cause of HF, n (%) |  |  |  |  |  |  |  |  |
| Ischemic | N/A | N/A | 11 (50) | 9 (47) | 10 (38) | 7 (27) | 6 (17) | 7 (29) |
| Nonischemic | N/A | N/A | 11 (50) | 10 (53) | 16 (62) | 19 (73) | 20 (83) | 17 (71) |
|  |  |  |  |  |  |  |  |  |
| Past medical history, n (%) |  |  |  |  |  |  |  |  |
| Hypertension | N/A | N/A | 14 (64) | 11 (58) | N/A | N/A | 8 (31) | 8 (33) |
| Coronary heart disease | 30 (73) | 27 (68) | 11 (50) | 10 (53) | 11 (42) | 13 (50) | 6 (17) | 7 (29) |
| Diabetes mellitus | N/A | N/A | 7 (32) | 4 (21) | N/A | N/A | N/A | N/A |
| Chronic lung disease | N/A | N/A | 6 (27) | 3 (16) | N/A | N/A | N/A | N/A |
| Stroke/TIA | N/A | N/A | 2 (9) | 2 (11) | N/A | N/A | N/A | N/A |
|  |  |  |  |  |  |  |  |  |
| NYHA functional class, n (%) |  |  |  |  |  |  |  |  |
| Ⅱ | N/A | N/A | 2 (9) | 2 (11) | 14 (54) | 13 (50) | 11 (43) | 12 (50) |
| Ⅲ | N/A | N/A | 20 (91) | 17 (89) | 12 (46) | 13 (50) | 15 (57) | 12 (50) |
| Ⅳ | N/A | N/A | 0 (0) | 0 (0) | 0 (0) | 0 (0) | 0 (0) | 0 (0) |
|  |  |  |  |  |  |  |  |  |
| Baseline 6-min walk, m | 269 ± 54 | 281 ± 44 | 317.5 ± 125.8 | 351.8 ± 117.1 | 416 ± 78 | 411 ± 109 | N/A | N/A |
| MLWHF score at baseline | 89 ± 12 | 89 ± 11 | 55.8 ± 19.8 | 59.2 ± 22.4 | 42 ± 23 | 49 ± 21 | N/A | N/A |
|  |  |  |  |  |  |  |  |  |
| Baseline ECG parameters |  |  |  |  |  |  |  |  |
| Heart rate, beats/min | 80 ± 12 | 82 ± 11 | 75 ± 15 | 72 ± 11 | 77 ± 9 | 81 ± 12 | N/A | N/A |
| QRS duration, ms | 92 ± 9 | 90 ± 10 | 105 ± 14 | 101 ± 14 | 119 ± 19 | 113 ± 21 | N/A | N/A |
|  |  |  |  |  |  |  |  |  |
| Baseline morphology |  |  |  |  |  |  |  |  |
| LVEF, % | 27 ± 8 | 29 ± 7 | 36.1 ± 11.9 | 42.9 ± 9.6 | 22 ± 8 | 25 ± 7 | 31.8 ± 7.7 | 33.7 ± 12.1 |
| Left atrial diameter, mm | 49 ± 5 | 47 ± 6 | N/A | N/A | 50 ± 6 | 46 ± 7 | 52 ± 11 | 50 ± 10 |
|  |  |  |  |  |  |  |  |  |
| Medical treatment, n (%) |  |  |  |  |  |  |  |  |
| ACEI/ARB | N/A | N/A | 21 (95) | 18 (95) | 25 (96) | 26 (100) | N/A | N/A |
| β blocker | N/A | N/A | 18 (82) | 18 (95) | 24 (92) | 24 (92) | N/A | N/A |
| Aldosterone antagonists | N/A | N/A | 10 (45) | 3 (16) | 13 (50) | 6 (23) |  |  |

Continued

|  | Biase et al. (5), 2016 | | Prabhu et al. (6), 2017 | | Marrouche et al. (7), 2018 | |
| --- | --- | --- | --- | --- | --- | --- |
|  | Ablation (n=102) | Medical therapy^#^ (n=101) | Ablation (n=33) | Medical rate control (n=33) | Ablation (n=179) | Medical therapy† (n=184) |
| Age, yrs | 62 ± 10 | 60 ± 11 | 59 ± 11 | 62 ± 9.4 | 64 (56-71) | 64 (56-73.5) |
| Male, n (%) | 77 (75) | 74 (73) | 31 (94) | 29 (88) | 156 (87) | 155 (84) |
| Body mass index, kg/m^2^ | 30 ± 8 | 29 ± 4 | 30 ± 7.5 | 31 ± 4.1 | 29.0 (25.9-32.2) | 29.1 (25.9-32.3) |
|  |  |  |  |  |  |  |
| Type of AF, n (%) |  |  |  |  |  |  |
| Paroxysmal | 0 (0) | 0 (0) | 0 (0) | 0 (0) | 54 (30) | 64 (35) |
| Persistent or long standing persisitent | 102 (100) | 101 (100) | 33 (100) | 33 (100) | 125 (70) | 120 (65) |
|  |  |  |  |  |  |  |
| Duration of AF, months | 8.6 ± 3.2 | 8.4 ± 4.1 | 23 ± 18 | 21 ± 15 | N/A | N/A |
|  |  |  |  |  |  |  |
| Cause of HF, n (%) |  |  |  |  |  |  |
| Ischemic | 63 (62) | 66 (65) | 0 (0) | 0 (0) | 72 (40) | 96 (52) |
| Nonischemic | 39 (38) | 35 (35) | 33 (100) | 33 (100) | 107 (60) | 88 (48) |
|  |  |  |  |  |  |  |
| Past medical history, n (%) |  |  |  |  |  |  |
| Hypertension | 46 (45) | 48 (48) | 13 (39) | 12 (36) | 129 (72) | 136 (74) |
| Coronary heart disease | 63 (62) | 66 (65) | 0 (0) | 0 (0) | 48 (27) | 66 (36) |
| Diabetes mellitus | 22 (22) | 24 (24) | 4 (12) | 5 (15) | 43 (24) | 67 (36) |
| Chronic lung disease | 46 (45) | 48 (48) | 12 (36) | 7 (21) | N/A | N/A |
| Stroke/TIA | N/A | N/A | 2 (6.1) | 0 (0) | 21 (12) | 21 (11) |
|  |  |  |  |  |  |  |
| NYHA functional class, n (%) |  |  |  |  |  |  |
| Ⅱ | N/A | N/A | 2.55 ± 0.62 | 2.45 ± 0.56 | 101 (58) | 109 (61) |
| Ⅲ | N/A | N/A |  |  | 50 (29) | 49 (27) |
| Ⅳ | N/A | N/A |  |  | 3 (2) | 2 (1) |
|  |  |  |  |  |  |  |
| Baseline 6-min walk, m | 348 ± 111 | 350 ± 130 | 491 ± 147 | 489 ± 132 | N/A | N/A |
| MLWHF score at baseline | 52 ± 24 | 50 ± 27 | N/A | N/A | N/A | N/A |
|  |  |  |  |  |  |  |
| Baseline ECG parameters |  |  |  |  |  |  |
| Heart rate, beats/min | N/A | N/A | 79 ± 17 | 77 ± 19 | N/A | N/A |
| QRS duration, ms | N/A | N/A | N/A | N/A | N/A | N/A |
|  |  |  |  |  |  |  |
| Baseline morphology |  |  |  |  |  |  |
| LVEF, % | 29 ± 5 | 30 ± 8 | 32 ± 9.4 | 34 ± 7.8 | 32.5 (25.0-38.0) | 31.5 (27.0-37.0) |
| Left atrial diameter, mm | 47 ± 4.2 | 48 ± 4.9 | 48 ± 5.5 | 47 ± 8.2 | 48.0 (45.0-54.0) | 49.5 (5.0-55.0) |
|  |  |  |  |  |  |  |
| Medical treatment, n (%) |  |  |  |  |  |  |
| ACEI/ARB | 94 (92) | 89 (88) | 31 (94) | 31 (94) | 166 (94) | 164 (91) |
| β blocker | 78 (76) | 81 (80) | 32 (97) | 32 (97) | 165 (93) | 171 (95) |
| Aldosterone antagonists | 46 (45) | 51 (50) | 11 (33) | 16 (48) | 165 (93) | 167 (93) |

* Rate control by atrioventricular node ablation with biventricular pacing.

# Medical therapy with amiodarone.

† Medical therapy with rate control or rhythm control strategies.

Values are mean ± standard deviation or median (interquartile range), unless otherwise stated.

ACEI, angiotensin converting enzyme inhibitor; AF, atrial fibrillation; ARB, angiotensin receptor inhibitor; ECG, electrocardiogram; HF, heart failure; LVEF, left ventricular ejection fraction; MLWHF, Minnesota Living with Heart Failure Questionnaire; N/A, not available; NYHA: New York Heart Association; TIA, transient ischemic attack.

**References**

1. Khan M, Jais P, Cummings J, Di Biase L, Sanders P, Martin D et al. Pulmonary-vein isolation for atrial fibrillation in patients with heart failure. *N Engl J Med* 2008; **17**:1778-85.

2. MacDonald M, Connelly D, Hawkins N, Steedman T, Payne J, Shaw M et al. Radiofrequency ablation for persistent atrial fibrillation in patients with advanced heart failure and severe left ventricular systolic dysfunction: a randomised controlled trial. *HEART* 2011; **9**:740-47.

3. Jones D, Haldar S, Hussain W, Sharma R, Francis D, Rahman-Haley S et al. A randomized trial to assess catheter ablation versus rate control in the management of persistent atrial fibrillation in heart failure. *J AM COLL CARDIOL* 2013; **18**:1894-903.

4. Hunter RJ, Berriman TJ, Diab I, Kamdar R, Richmond L, Baker V et al. A randomized controlled trial of catheter ablation versus medical treatment of atrial fibrillation in heart failure (the CAMTAF trial). *Circ Arrhythm Electrophysiol* 2014; **1**:31-38.

5. Di Biase L, Mohanty P, Mohanty S, Santangeli P, Trivedi C, Lakkireddy D. Ablation Versus Amiodarone for Treatment of Persistent Atrial Fibrillation in Patients With Congestive Heart Failure and an Implanted Device: Results From the AATAC Multicenter Randomized Trial. *CIRCULATION* 2016; **17**:1637-44.

6. Prabhu S, Taylor AJ, Costello BT, Kaye DM, McLellan A, Voskoboinik A. Catheter Ablation Versus Medical Rate Control in Atrial Fibrillation and Systolic Dysfunction: The CAMERA-MRI Study. *J AM COLL CARDIOL* 2017; **16**:1949-61.

7. Marrouche NF, Brachmann J, Andresen D, Siebels J, Boersma L, Jordaens L. Catheter Ablation for Atrial Fibrillation with Heart Failure. *N Engl J Med* 2018; **5**:417-27.
